# Salivary Heparanase Level Is a Potential Biomarker to Diagnose and Prognose the Malignant Salivary Gland Tumor

**DOI:** 10.1371/journal.pone.0143009

**Published:** 2015-11-16

**Authors:** Xiangbing Wu, Jun Yu, Guilin Gao, Xin Wang, Yang Liu, Shengrong Zhu, Zhongjian Gong

**Affiliations:** 1 Department of Stomatology, Wuxi People’s Hospital, Nanjing Medical University, Wuxi, China; 2 Center of Stomatology, Central Hospital of Enshi Autonomous Prefecture, Enshi, China; 3 Center of Stomatology, Tongji Hospital, Tongji Medical College, Huazhong University of Science and Technology, Wuhan, China; Sapporo Medical University, JAPAN

## Abstract

**Background:**

Upregulation of heparanase has been reported in an increasing number of human cancer tissues. However, the level of salivary heparanase and its clinical significance in patients with salivary gland tumors remain unclear.

**Methods:**

Salivary heparanase levels in patients with salivary gland tumors were detected using enzyme-linked immunosorbent assays (ELISAs) and the clinical significance was evaluated by analyzing the correlations among salivary heparanase levels, clinicopathological parameters, and clinical outcomes.

**Results:**

The levels of salivary heparanase were significantly higher in patients with malignant salivary gland tumors than in benign tumors and normal controls (P<0.0001). High salivary heparanase levels were positively correlated with increased lymph node metastasis (P = 0.0235) and poorer tumor node metastasis stage (TNM) (P = 0.0183). Survival analyses revealed that high salivary heparanase levels were associated with worse overall survival (P = 0.0023) and disease-free survival (DFS) (P = 0.0025).

**Conclusions:**

The study shows that salivary heparanase levels, as detected by the ELISAs, can be used to diagnose and provide an accurate prognosis for malignant salivary gland tumors. Salivary heparanase level was an independent predictor in patients with malignant salivary gland tumors.

## Introduction

Salivary gland tumors, characterized by widely varied phenotypic features and unpredictable clinical outcomes, account for approximately 3–6% of all tumors of the head and neck [1]. The overlapping histology and variable biological progression pose challenges for differentiating benign and malignant salivary gland tumors. Despite continuous efforts to identify new approaches to improve treatment and diagnosis, progress has been unsatisfactory in the past three decades. Therefore, the application of novel and effective biomarkers and methods for diagnosing and predicting salivary gland tumors are still needed.

Heparanase is an endoglycosidase capable of cleaving heparan sulfate (HS) side chains of heparan sulfate proteoglycans (HSPG), major constituents of the extracellular matrix (ECM) [2]. Because the ECM provides an essential physical barrier between cells and tissues, heparanase activity has long been correlated with the metastatic potential of cancer cells [3]. Heparanase is also implicated in inflammation and angiogenesis as a consequence of HS cleavage, which can release many kinds of biological mediators such as growth factors, cytokines, and chemokines in response to local or systemic signals [4–6]. Recent studies have shown that heparanase of tumor tissues, blood, or urine is closely linked with an increasing number of human cancer types, including pancreatic [7], gastric [8], bladder [9], colon [10], and cervical cancer [11]. Heparanase upregulation of tumor tissues, blood, or urine have been correlated with increased lymph node and distant metastasis and with shorter postoperative survival of cancer patients. Therefore, heparanase is an important biomarker for diagnosis, therapy, and prognosis of some hematological and solid tumors. However, heparanase levels in saliva and tumor tissues of patients with salivary gland tumors and its clinical significance have not been reported.

In our current study, we confirmed that the levels of heparanase in saliva and tumor tissues were significantly higher in patient with malignant salivary gland tumors than in benign tumors and normal controls, by using immunochemistry and ELISAs. Furthermore, we evaluated the clinical significance of salivary heparanase levels and found that high levels of heparanase in saliva are notably associated with increased lymph node metastasis and poorer TNM stage in patients with malignant salivary gland tumors. In addition, we also demonstrated that patients whose saliva contained high levels of heparanase had worse overall survival and disease-free survival, and that salivary heparanase level was an independent prognostic factor in patients with malignant salivary gland tumors.

## Methods

### Patients and specimens

Saliva and tumor tissue specimens were obtained from a cohort of 126 patients who were histologically diagnosed with salivary gland tumors (59 patients with benign tumors and 67 patients with malignant tumors) and underwent initial surgical treatment in the Department of Stomatology, Wuxi People’s Hospital, Nanjing Medical University, Wuxi, China and the Center of Stomatology, Tongji Hospital, Tongji Medical College, Huazhong University of Science and Technology, Wuhan, China, between December 2006 and January 2010. All tumor tissues were embedded in paraffin for pathological examinations and immunohistochemical staining. The saliva of patients was collected under non-stimulatory conditions between 9 AM and 11 AM before the surgical treatment. Patients were asked to rinse their mouth with sterilized purified water before generating saliva and then spit saliva into a centrifuge tube until 5 ml of saliva was collected. Saliva obtained from 25 healthy donors, matched by age and sex, was used as the normal control, which was collected with the same conditions and methods. Saliva was immediately centrifuged (4°C; 1000 × *g*; 15 min) after collection. Precipitates which contained squamous cells and cell debris were removed and supernatants were quickly frozen at –80°C until the ELISAs were performed. The medical records of the patients were retrospectively reviewed for inclusion and exclusion criteria, which were collected in March 2015. The authors had access to identifying information during or after data collection. Exclusion criteria included chemotherapy and/or preoperative radiotherapy, recurrent tumors, distant metastasis, and/or incomplete medical records. All 67 patients with malignant tumors had clinical information and follow-up data, and the median follow-up time was 64 months. The clinicopathological characteristics of 67 patients are summarized in S1 Table. The study was approved by the Institutional Ethics Committee of Wuxi People’s Hospital, Nanjing Medical University (number: 2007051) and Tongji Hospital, Tongji Medical College, Huazhong University of Science and Technology (number: 2006–11). All of the patients and healthy donors signed written informed consent in accordance with the institutional guidelines. The study conformed to the tenets of the Declaration of Helsinki.

### Immunohistochemical analysis

Immunohistochemical staining was performed using a rabbit polyclonal heparanase antibody (1:200; Abcam, Cambridge, MA, USA). Briefly, paraffin-embedded tissue samples were cut into 4 μm tissue sections and were deparaffinized in xylene, rehydrated in graded ethanol, and treated with citrate buffer for heat-induced epitope retrieval. Tissue sections were incubated with 2.5% normal goat serum for 40 minutes to block nonspecific binding, and incubated with rabbit polyclonal heparanase antibody (4°C; overnight), followed by incubation with a biotinylated secondary antibody and ABC reagent, using the manufacturer’s instructions (Vector Laboratories, Burlingame, CA, USA). The labeling index was defined semi-quantitatively as the intensity of staining (0, 1, 2, or 3) multiplied by the percentage of positive cells.

### ELISA analysis

The levels of heparanase in saliva were detected following the manufacturer’s instructions for the Human Heparanase ELISA Kit (EIAab, Wuhan, China). Briefly, 40 ul of saliva supernatant and 10 ul of biotinylated heparanase antibody were added into each well of an assay plate, mixed gently, and incubated for 1 hour at 37°C. Then, 50 ul of horse-radish peroxidase (HRP) conjugated reagent were added and incubated for 1 hour at 37°C. The reaction was visualized by the addition of 50 μl of chromogenic substrate for 10 min at 37°C in dark conditions, and stopped with 50 μl of stop solution. The optical density of each well was measured with a microplate reader at 450 nm. Each well of the assay plate was aspirated and washed with wash buffer for five times after each step. As a reference for quantification, serial dilutions of standards were added into the assay plate at the same time, and a standard curve was established. Measurements were performed in triplicate. The median was set as the cut-off value to determine high or low levels of heparanase in saliva. Regarding the patients with malignant salivary gland tumors, the cut-off value was 377 (range 113–743), a value >377 was considered high salivary heparanase level, and a value ≤377 was considered low salivary heparanase level.

### Statistical analysis

Student’s *t*-tests were used to analyze the difference of heparanase levels in saliva and tumor tissues between patients with malignant salivary gland tumors, benign tumors, and healthy donors. The consistency of heparanase level detected by immunochemistry and ELISAs were evaluated using Pearson’s correlation analyses. The associations between salivary heparanase levels and patient characteristics were analyzed by Student’s *t*-test (2 subgroups) and by one-way ANOVA (>2 subgroups). The log-rank test was used to analyze univariate associations between salivary heparanase levels and overall survival and disease-free survival. The Cox proportional hazards model was used for multivariate analyses and all potential prognostic factors with P values <0.05 from the univariate analyses were incorporated into multivariate analyses. All tests were two-sided, and P values less than 0.05 were considered statistically significant.

## Results

### Heparanase levels in saliva and tumor tissue specimens of patients with salivary gland tumors

**Fig 1:**
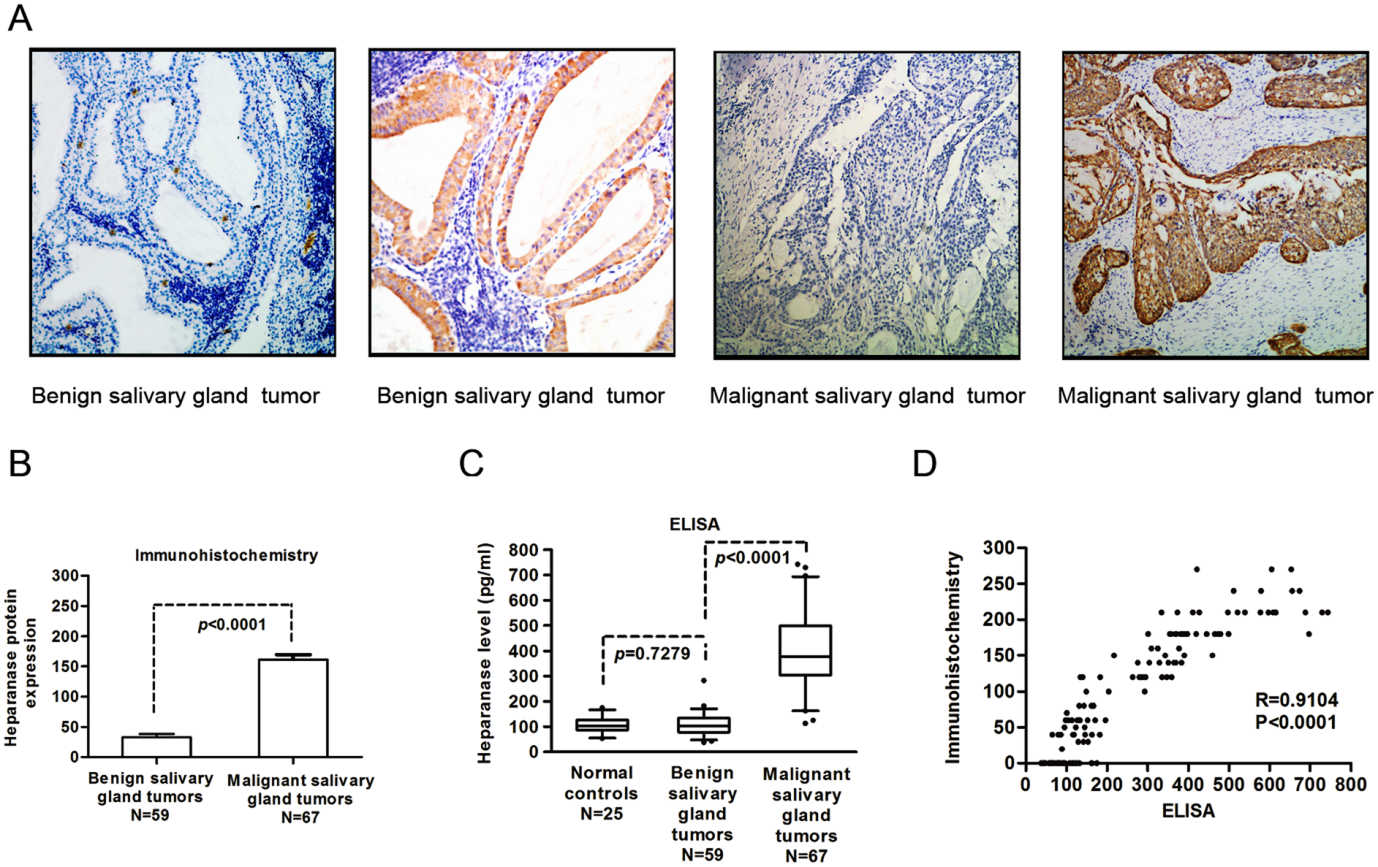
Heparanase levels in saliva and tumor tissue specimens were investigated with immunohistochemical staining and an ELISAs. (A) Representative images show heparanase-negative and heparanase-positive immunohistochemical staining in benign and malignant salivary gland tumors. (B) Heparanase levels in 59 benign salivary gland tumor tissues and 67 malignant tumor tissues were evaluated with immunohistochemical staining. (C) Salivary heparanase levels of 59 patients with benign salivary gland tumors, 67 patients with malignant tumors, and 25 healthy donors (normal controls) were determined using ELISAs. (D) The consistency of heparanase levels in saliva and tumor tissues from 126 patients with salivary gland tumors (59 patients with benign tumors and 67 patients with malignant tumors) were evaluated using Pearson’s correlation analysis.

To determine heparanase levels, immunohistochemical staining was first performed to detect the heparanase protein level in 67 malignant salivary gland tumors and 59 benign tumors. Heparanase expression was observed as cytoplasmic staining. The intensity of heparanase expression in salivary gland tumors ranged from negative to strongly positive, and the extent of heparanase expression varied from no tumor cells having been stained to more than 90% stained tumor cells. Representative photomicrographs of heparanase-negative and heparanase-positive staining are provided in Fig 1 panel A. The levels of heparanase in malignant salivary gland tumor tissues were significantly higher than that in benign salivary gland tumor tissues (P<0.0001, Fig 1 panel B). In an attempt to find a more simple and sensitive method to differentiate malignant salivary gland tumors from benign tumors, the level of heparanase in saliva was determined by an ELISAs. As is shown in Fig 1 panel C, salivary heparanase levels of patients with malignant salivary gland tumors were significantly higher than that of patients with benign tumors and that of normal controls (P<0.0001). Nevertheless, differences of salivary heparanase levels were not found between patients with benign salivary gland tumors and normal controls (P = 0.7279). To evaluate the reliability and accuracy of this new method, Pearson’s correlation analyses were performed to estimate the consistency of heparanase levels detected by these two methods in the same cohort of 126 patients. A significantly positive correlations between the results of immunochemistry and the ELISAs was observed (Pearson’s correlation, R = 0.9104, P<0.0001) (Fig 1 panel D).

### Correlations between salivary heparanase levels and clinicopathological parameters in patients with malignant salivary gland tumors

**Fig 2:**
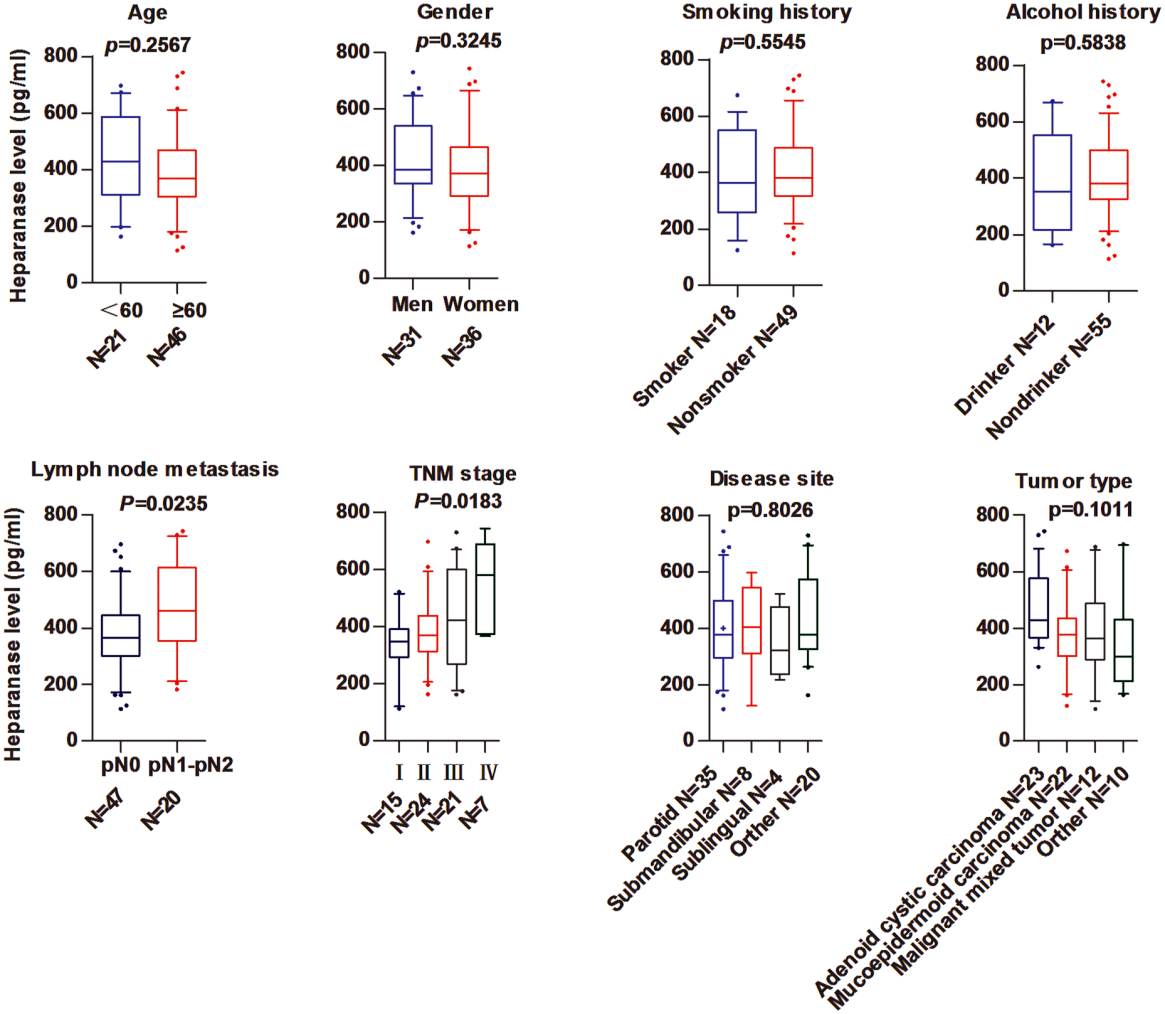
Correlations between salivary heparanase levels and clinicopathological parameters in patients with malignant salivary gland tumors. The associations between salivary heparanase levels and clinicopathological parameters—age, gender, smoking history, alcohol history, lymph node metastasis, TNM stage, disease site, and tumor type—were analyzed in patients with malignant salivary gland tumors.

By using Student’s *t*-test (2 subgroups) and one-way ANOVA (>2 subgroups) to analyze the associations between salivary heparanase levels and clinicopathologic parameters in patients with malignant salivary gland tumors, we found that high salivary heparanase levels were correlated with increased lymph node metastasis (P = 0.0235) and poorer TNM stage (P = 0.0183), whereas no significant associations were determined between salivary heparanase levels and age (P = 0.2567), gender (P = 0.3245), smoking history (P = 0.5545), alcohol history (P = 0.5838), disease site (P = 0.8026), or tumor type (P = 0.1011) (Fig 2).

### Associations between salivary heparanase levels and clinical outcomes in patients with malignant salivary gland tumors

**Fig 3:**
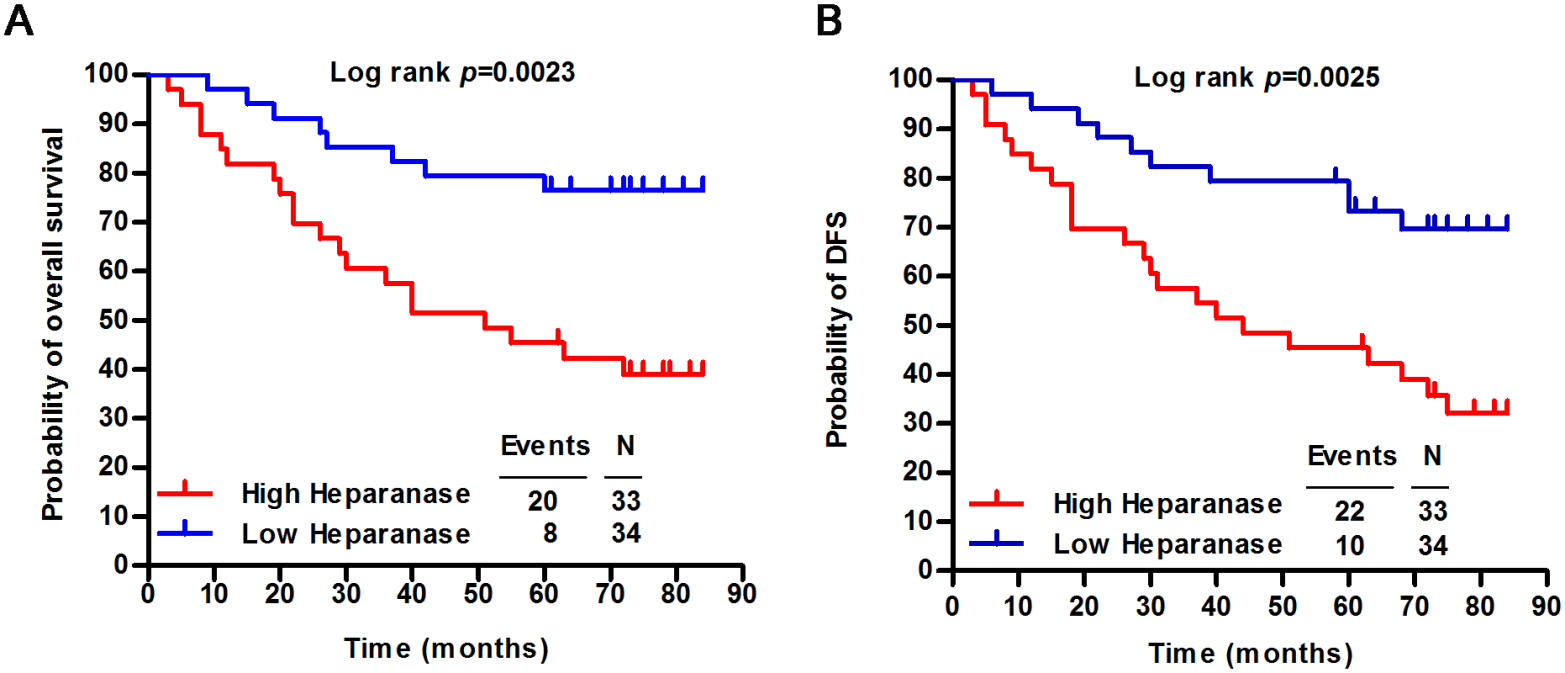
Correlations between salivary heparanase levels and clinical outcomes in patients with malignant salivary gland tumors. Kaplan-Meier survival curves show the (A) overall survival, and (B) disease-specific survival of patients with malignant salivary gland tumors relative to salivary heparanase levels is correlated.

To investigate the correlations between salivary heparanase levels and clinical outcomes, overall survival and the disease-free survival probability were evaluated by using Kaplan-Meier survival analyses. We observed that 76% (26 of 34) of the patients whose saliva contained low levels of heparanase were still alive at 84 months, whereas only 39% (13 of 33) of the patients with high levels of salivary heparanase were alive at 84 months (P = 0.0023, Fig 3 panel A). In addition, the disease-specific survival rates of patients with low levels of salivary heparanase were significantly higher than those of patients with high levels of salivary heparanase (P = 0.0025) (Fig 3 panel B). In univariate and multivariate Cox proportional analyses (Table 1), salivary heparanase level [hazard ratio, 2.805; 95% confidence interval (CI), 1.218–6.460; P = 0.015] was identified as an independent predictor of clinical outcome in patients with malignant salivary gland tumors.

**Table 1:** Univariate and Multivariate Cox Proportional Hazards Regression Models for Estimating Overall Survival.

Characteristics	HR	95%CI	P
**Univariate analysis**			
Overall survival			
Age (<60 y vs ≥ 60 y)	0.533	0.252–1.122	0.098
Sex (men vs women)	0.942	0.448–1.980	0.874
Smoking history (smoker vs nonsmoker)	0.683	0.277–1.687	0.409
Alcohol history (drinker vs nondrinker)	0.689	0.239–1.986	0.49
Tumor type	0.798	0.562–1.132	0.205
TNM stage	2.55	1.619–4.016	0.001
Lymph node metastasis (pN0 vs pN1-pN2)	4.613	2.168–9.814	0.001
Heparanase level (high vs low)	3.319	1.459–7.549	0.004
Disease site	0.846	0.552–1.296	0.442
**Multivariate analysis**			
Overall survival			
TNM stage	1.736	0.930–3.242	0.083
Lymph node metastasis (pN0 vs pN1-pN2)	2.176	0.753–6.284	0.151
Heparanase level (high vs low)	2.805	1.218–6.460	0.015

Abbreviations: CI, confidence interval; HR, hazard ratio; pN, pathological lymph node status; TNM, tumor-lymph node-metastasis

## Discussion

The limitations of conventional methods for diagnosing and predicting salivary gland tumors have necessitated a search for novel potential approaches. Recently, an increasing number of genes and signaling molecules were reported to be involved in the initiation and progression of salivary gland tumors [12–15]. It has been demonstrated that heparanase of tumor tissues was upregulated in 70% of malignant salivary tumors and was inversely correlated with cumulative survival and disease-free survival [16]. This result suggests that heparanase may act as a potential and novel biomarker for early diagnosis and prognosis of salivary gland tumors. In our study, we first investigated the levels of heparanase in salivary gland tumor tissues with immunohistochemical staining and found that the heparanase levels in malignant salivary tumor tissues were significantly higher than in benign tumor tissues. Although immunohistochemical staining, as a classical and traditional diagnostic tool, has an irreplaceable function for identifying the intricate histology of salivary gland tumors, simpler and more sensitive approaches are also needed.

It has been reported that ELISAs can quantify the heparanase levels in body fluids. Urinary heparanase levels, determined by this sensitive and simple method, were markedly elevated in patients with leukemia and bladder carcinoma [17]. Saliva, a secretion of the salivary glands, has long been regarded as a useful biological fluid for analyzing electrolytes, hormones, RNA, antibodies, enzymes, and a variety of other proteins [18–22]. Because saliva testing has its own unique advantages, such as noninvasiveness, ease of collection, affordability, and safety, compared to blood testing and other types of diagnostic methods, it has been utilized to diagnose numerous disease states such as allergies, human immunodeficiency virus (HIV) infection, periodontitis, hypogonadism, and cardiovascular disease [23–30]. Zhang et al. reported that the combination of four mRNA biomarkers—KRAS, MBD3L2, ACRV1, and DPM1—in saliva supernatant could differentiate pancreatic cancer patients from non-cancer subjects [31]. Streckfus et al. reported that salivary levels of c-erbB-2 were significantly higher in women with breast cancer than in women with benign breast lesions and healthy women [32]. Nagler et al. demonstrated, using ELISAs, that salivary heparanase levels of patients with tongue cancer were nearly three times as high as those of healthy controls [33]. However, the variations of salivary heparanase levels in patients with salivary gland tumors and the clinical significances have never been reported. In this study, we confirmed that the salivary heparanase levels in patients with malignant salivary gland tumors were significantly higher than those of patients with benign tumors and of healthy donors. This finding was consistent with the results of immunohistochemical staining, and Pearson’s correlation analysis also confirmed the consistency of results detected by these two methods. Furthermore, we evaluated the clinical significance of salivary heparanase levels by analyzing the correlations between the salivary levels of heparanase and clinicopathological parameters and clinical outcomes in patients with malignant salivary gland tumors. Our data showed that the high levels of salivary heparanase were associated with increased lymph node metastasis (P = 0.0235) and poorer TNM stage (P = 0.0183). Moreover, we also observed that patients whose saliva contained high levels of heparanase had worse overall survival (P = 0.0023) and disease-specific survival (P = 0.0025) and that salivary heparanase levels, by univariate and multivariate analyses, were independent prognostic factors in patients with malignant salivary gland tumors (hazard ratio, 2.805; 95% CI, 1.218–6.460; P = 0.015).

## Conclusions

In this study, we found that the levels of salivary heparanase increased in patients with malignant salivary gland tumors, and that high salivary heparanase levels were positively correlated with increased lymph node metastasis, poorer TNM stage, worse overall survival, and disease-free survival in patients with malignant salivary gland tumors. Taken together, our study indicates that salivary heparanase level may serve as a potential biomarker to diagnose and predict malignant salivary gland tumors.

## Supporting Information

S1 Table Clinicopathologic Characteristics of 67 Patients with Malignant Salivary Gland Tumor. (DOC)
