# Supplementary material for: Salivary Heparanase Level Is a Potential Biomarker to Diagnose and Prognose the Malignant Salivary Gland Tumor
Source: PLoS One. 2015 Nov 16;10(11):e0143009. doi: 10.1371/journal.pone.0143009 (PMC4646693; doi:10.1371/journal.pone.0143009)
Supplement: S1 Table — (DOC) [file pone.0143009.s001.doc]

Table S1 Clinicopathologic Characteristics of 67 Patients with Malignant Salivary Gland Tumor

| Characteristic | Classification | Case number | Percentage (%) |
| --- | --- | --- | --- |
| Age, years | <60 | 21 | 31.3 |
|  | ≥60 | 46 | 68.7 |
| Sex | Men | 31 | 46.3 |
|  | Women | 36 | 53.7 |
| Alcohol history | Drinker | 12 | 17.9 |
|  | Nondrinker | 55 | 82.1 |
| Smoking history | Smoker | 18 | 26.9 |
|  | Nonsmoker | 49 | 73.1 |
| Tumor type | Adenoid cystic carcinoma | 23 | 34.3 |
|  | Mucoepidermoid carcinoma | 22 | 32.8 |
|  | Malignant mixed tumor | 12 | 17.9 |
|  | Orther | 10 | 14.9 |
| TNM stage | I | 15 | 22.4 |
|  | II | 24 | 35.8 |
|  | III | 21 | 31.3 |
|  | IV | 7 | 10.4 |
| Lymph node metastasis | pN0 | 47 | 70.1 |
|  | pN1-pN2 | 20 | 29.9 |
| Disease site | Parotid | 35 | 52.2 |
|  | Submandibular | 8 | 11.9 |
|  | Sublingual | 4 | 6.0 |
|  | Orther | 20 | 29.9 |
